# A Comparative Analysis of Telerehabilitation and Telemedicine Utilization During the COVID-19 Pandemic in Poland: Trends, Patterns, and Implications

**DOI:** 10.5195/ijt.2024.6627

**Published:** 2024-06-28

**Authors:** Anna Krawczyk, Mikołaj Marszałek

**Affiliations:** 1 Department of Innovation in Healthcare, Warsaw School of Economics, Warsaw, Poland; 2 Department of Social Medicine and Public Health, Medical University of Warsaw, Warsaw, Poland; 3 Medical University of Warsaw, Warsaw, Poland

**Keywords:** Ambulatory care, COVID-19, Telehealth utilization, Telerehabilitation

## Abstract

The study aimed to examine the influence of COVID-19 on the adoption of teleservices in Poland, with a focus on contrasting patterns between rehabilitation and ambulatory care settings. We conducted a retrospective analysis of a national dataset to assess trends in telehealth use from 2020 to 2022. The use of teleservices peaked in April 2020 in both sectors. The share of the teleservices in the period of October 2020-December 2022 was much higher in the ambulatory (average 7,8%) than the rehabilitation sector (average 0,16%). Although, the analysis showed a moderate relationship between COVID-19 incidence and telehealth utilization (Spearman's rho from 0.39 and 0.52). Our findings demonstrate no statistically significant difference in Spearman's rho values between ambulatory care and rehabilitation, indicating a similar strength of response to the pandemic waves. Our findings underscore the importance of telehealth services in ensuring healthcare accessibility during times of crisis, emphasizing their role in facilitating continuity of care amidst pandemic-related disruptions. This study contributes to the understanding of telehealth utilization trends during the COVID-19 pandemic, offering insights into the adaptive responses of healthcare systems to unprecedented challenges. Further research is warranted to explore the long-term implications of telehealth use and to inform strategies for optimizing healthcare delivery in post-pandemic contexts.

## Introduction

Amid the widespread adoption of telehealth modalities, gaining insights into the patterns and trends underlying their use becomes imperative for guiding healthcare policy and resource allocation. Throughout the COVID-19 pandemic, healthcare providers have increasingly explored and expanded their understanding of telehealth capabilities (Colbert et al., 2020). Notably, telemedicine has emerged as an effective approach to managing COVID-19 (Mehraeen et al., 2023), with potential applications extending to post-COVID-19 patient management, as suggested by Bartczak et al. (2022).

### Telerehabilitation Regulations

In Poland, healthcare operates within an insurance-based framework administered by the National Health Fund (NFZ), functioning as a publicly funded healthcare system. Access to healthcare services is universally available at no cost to eligible Polish citizens falling under the “insured” category, typically requiring health insurance provided by an employer or being a dependent of an insured individual.

The provision of telerehabilitation services, under public funding, is regulated by Ministry of Health guidelines. Consequently, only hybrid telerehabilitation in cardiology is among the guaranteed services for beneficiaries in the realm of telerehabilitation. This service can be delivered within daycare centers or units, as well as in inpatient settings.

On April 10, 2020, the Ministry of Health introduced new regulations pertaining to the utilization of telemedicine and telerehabilitation services during states of epidemic threat or epidemic. These regulations allow for medical consultations, advice, or therapeutic visits to be conducted via teleinformatics or communication systems, provided that such methods do not endanger the health condition of the beneficiary.

As a result, between April 10, 2020, and July 1, 2023, ambulatory and homecare telerehabilitation, along with other telehealth services, were eligible for insurance coverage.

### Patterns of COVID-19 Incidence in Poland

**Figure 1:**
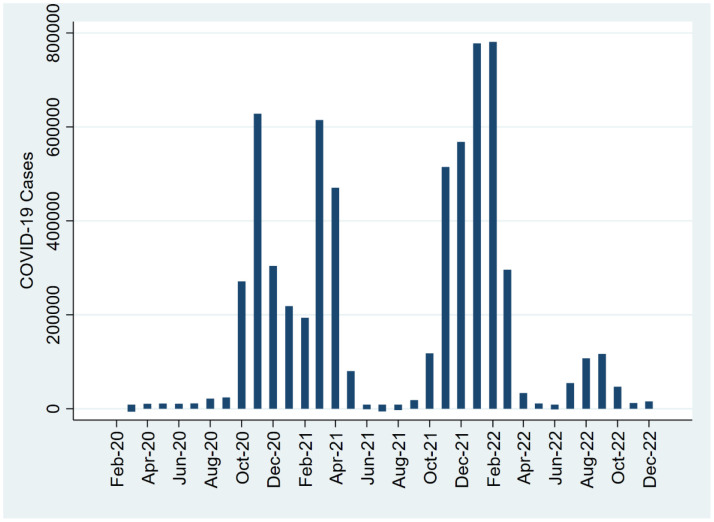
COVID-19 Incidence in Poland

Comprehending the dynamics of the pandemic is imperative for further analysis. Figure 1 illustrates monthly COVID-19 case data from January 2020 to December 2022, forming the basis of our investigation into the temporal dynamics of the pandemic in Poland.

Our analysis divulges distinct patterns in reported cases over time. Initially, a period of low case numbers characterized early 2020, followed by a significant surge during the first wave of the pandemic from April to June 2020, peaking in May 2020. Subsequently, a second wave emerged in late 2020, reaching its apex in November 2020. Throughout 2021 and 2022, case numbers exhibited fluctuation, indicative of the persistent challenges posed by the pandemic. These trends speak to the dynamic nature of COVID-19 transmission and emphasize the importance of continuous surveillance and public health interventions. To facilitate interpretation of our findings, we included a plot of monthly COVID-19 cases distribution over the study period.

## Methods

An observational retrospective study was conducted using national data from two sectors: rehabilitation and ambulatory care as control.

### Definitions

Ambulatory care - outpatient specialist care in all fields except rehabilitation and psychological and psychiatric services.

Rehabilitation care – outpatient/ambulatory services in rehabilitation (without home-care).

Total number of services - the number of unique identifiers of unit products delivered.

Number of teleservices - the number of unique identifiers of products for which the procedure that determines the provision of a service using ICT (Information and Communications Technology) systems or communication systems was reported. Identification of teleconsultations in the NHF service database is conducted based on the reported procedure codes according to the ICD-9 classification.

### Data Collection

The dataset was exported from the official national database “Maps of Health Needs” (Retrieved March 2024 from: https://basiw.mz.gov.pl/en/maps-of-health-needs/map-of-health-needs-for-the-period-2022-2026/analyses/4256-2/) by the Department of Analyses and Strategies of the Ministry of Health. The study period spans from January 2020 to December 2022.

Database is based on information reported by healthcare providers to the National Health Fund (NFZ), so the analyses only concern rehabilitation and other ambulatory services financed by the National Health Fund. In particular, services financed by the Social Insurance Institution (ZUS), the Agricultural Social Insurance Fund (KRUS) and from patients' private funds were not included. Patients from all age groups are included in the study. Home-care rehabilitation data was excluded.

The data about COVID-19 incidence was taken from Polish Ministry of Health reports, retrieved March 2024 from: https://koronawirusunas.pl/.

### Study Design

This study examined the monthly utilization of telerehabilitation services in Poland from January 2020 to December 2022, comparing it to the utilization of teleservices in ambulatory care. The relationship between telerehabilitation use and COVID-19 cases was investigated, with results compared to ambulatory care to assess any significant differences in correlation. Correlation analysis covers the period from October 2020 to December 2022, capturing the onset of the second wave of COVID-19 in Poland until the end of the study interval. This exclusion of the first wave of COVID-19 cases allows for a focused and contextually relevant examination of factors influencing COVID-19 dynamics during the specified timeframe. Analysis was conducted using Microsoft Excel, STATA SE 17, and R (R Core Team 2022).

## Results

**Figure 2:**
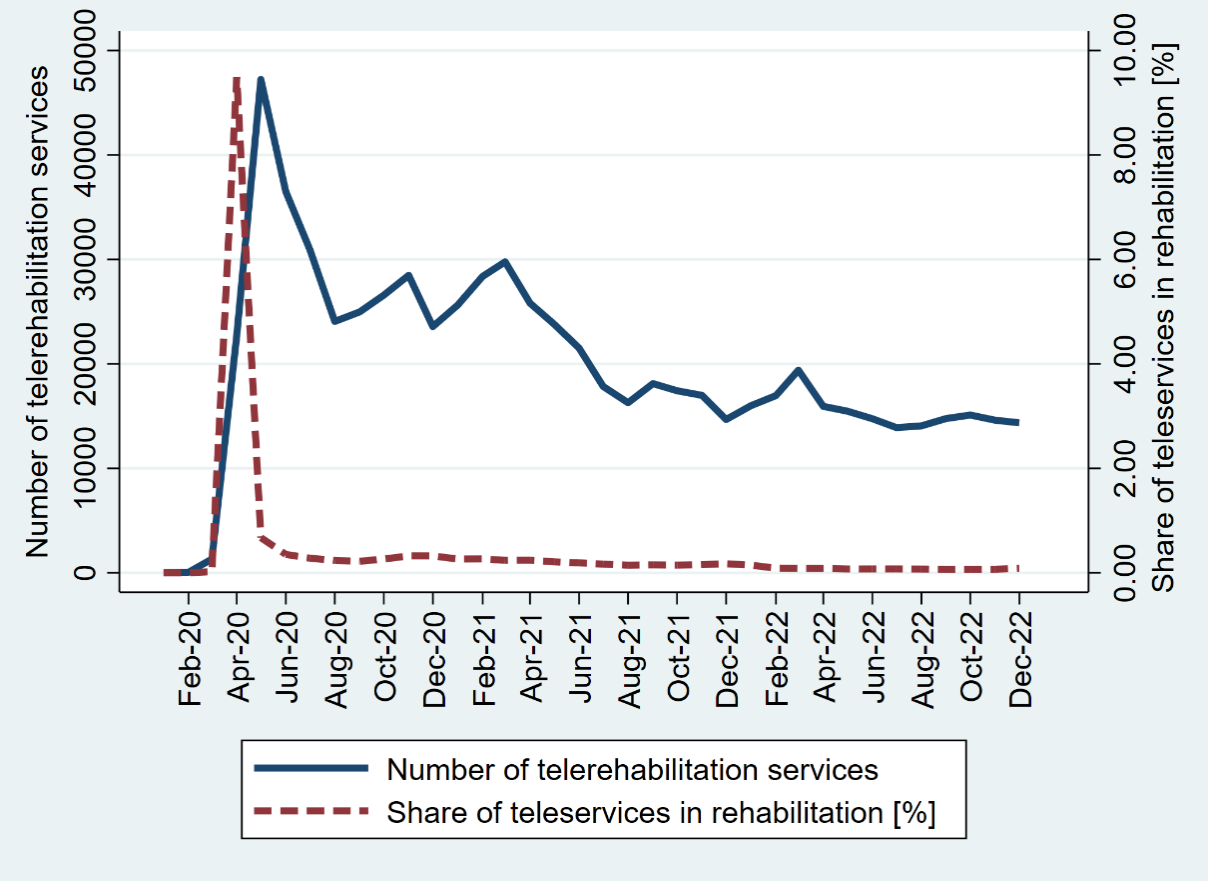
Monthly Distribution of Telerehabilitation Services and Share of Teleservices in the Rehabilitation Sector

**Figure 3:**
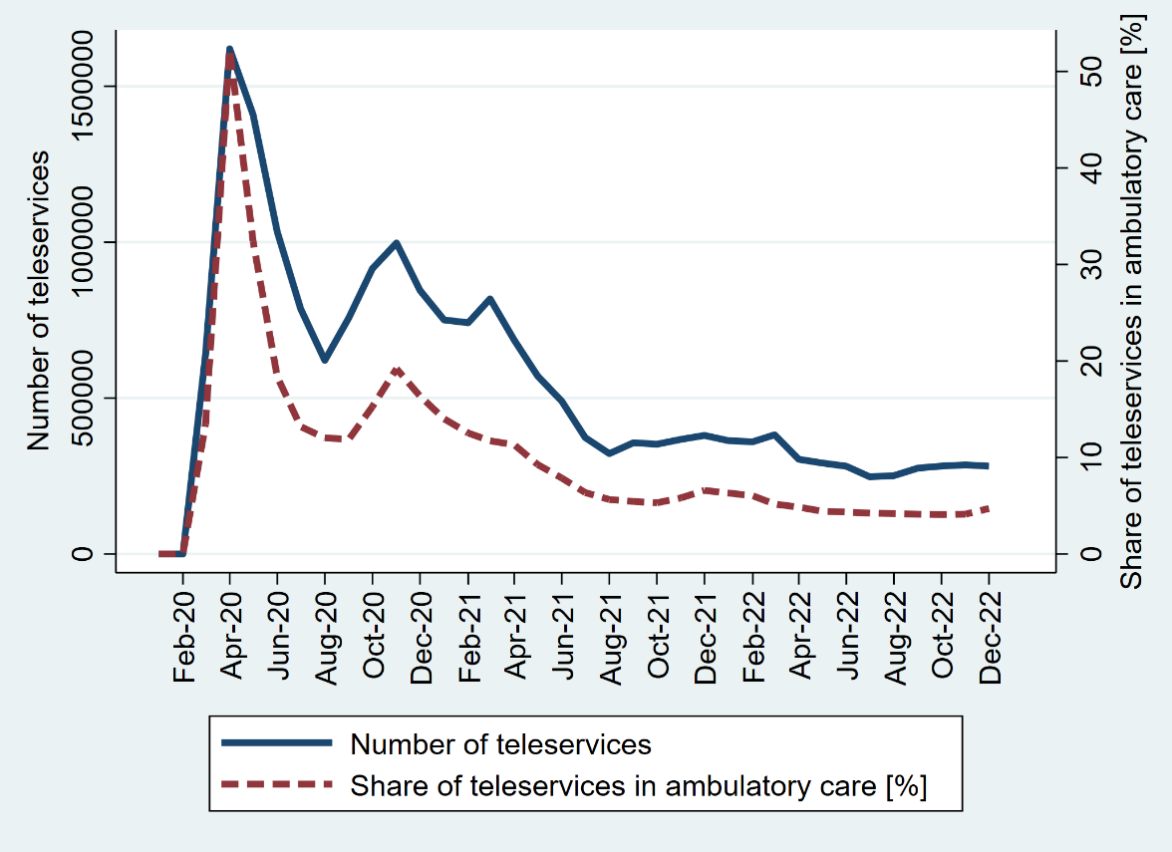
Monthly Distribution of Telemedicine Services and Share of Teleservices in Ambulatory Care

Over the study period, the total number of telerehabilitation services amounted to 708,042, while in the ambulatory sector tele-services totaled 19,440,707. The share of teleservices among all were much higher in the ambulatory (average 7,8%) than the rehabilitation sector (average 0,16%). The monthly distribution of the number of teleservices and the proportion of teleservices among all services is illustrated in Figure 2 for the rehabilitation sector and in Figure 3 for ambulatory care sector.

Focusing on the period from April 2020 to December 2022, which marks the phase when teleservices began to be widely financed by the public system and extends throughout the observation period, we observe significant trends both in rehabilitation and ambulatory care. Both the number of teleservices and the share of teleservices among all peaked in April 2020. Subsequently, teleservices utilization fluctuated over time but eventually stabilized. These interpretations highlight the transformative effect of the pandemic on healthcare delivery, particularly the rapid adoption and sustained telehealth use in both the telerehabilitation and ambulatory care settings.

The study employed Spearman rank correlation tests on monthly cross-sections to assess the relationship between COVID-19 cases and separately several variables: the number of telerehabilitation consultations (Telerehabilitation services), the number of teleconsultations in ambulatory care (Ambulatory teleservices), the percentage share of teleservices in rehabilitation (% telerehabilitation among all), and the percentage share of teleservices in ambulatory care (% ambulatory teleservices among all). Differences in Spearman's rho between groups were determined using bootstrapping and percentile estimation, with 999 replications for each comparison. Results are presented in Table 1.

**Table 1:** Result of the Spearman Rank Correlation between Monthly Number of COVID-19 Cases and Given Variables

Variable	Spearman's rho	95% Confidence Interval	p - value	Significance
Telerehabilitation services	0.3901	(0.0347, 0.6698)	0.0443	p<0.05
% telerehabilitation among all	0.4249	(0.0788, 0.6783)	0.0272	p<0.05
Ambulatory teleservices	0.5195	(0.2239, 0.7327)	0.0055	p<0.05
% ambulatory teleservices among all	0.4908	(0.2047, 0.7002)	0.0093	p<0.05

As the incidence of COVID-19 cases increased, a simultaneous rise in telehealth use was noted in both rehabilitation and ambulatory care settings. Spearman's rho values ranging between 0.39 and 0.52 indicate a moderate relationship between the variables. The significance of differences in rho between ambulatory care and rehabilitation was evaluated, with results presented in Table 2.

**Table 2:** Difference in Rho between Ambulatory Care and Rehabilitation

	Rho difference	95% Confidence Interval	p - value	Significance
Difference in number of teleservices	0.1294	−0.2851, 0.5342	0.51	p>0.05
Difference in share among all	0.0659	−0.3078, 0.4522	0.76	p>0.05

There was no statistically significant difference observed in Spearman's Rho values between Ambulatory Care and Rehabilitation, concerning both the number of teleservices and their share among all services. This suggests that rehabilitation and ambulatory care responded similarly, in terms of the strength of correlation, to the waves of COVID-19, as indicated by the number of new COVID-19 cases.

### Limitations

In this study, several limitations should be acknowledged. Firstly, the analysis is confined to publicly funded sectors, thereby excluding data from private healthcare services. The utilization patterns and dynamics of telerehabilitation in private healthcare settings may differ significantly from those observed in publicly funded sectors. Additionally, the study does not consider potential regional variations in healthcare infrastructure and access, which could impact the generalizability of findings across different geographical areas within Poland. Furthermore, the database in the time of study consisted only of data from 2020-2022, that is why it was chosen as the study period. The absence of pre-pandemic data on telerehabilitation use limits the ability to compare trends and assess the true impact of COVID-19 on the adoption and utilization of telerehabilitation services in Poland.

## Discussion

The findings of this study underscore the complex interplay between telerehabilitation (TR) utilization and epidemiological factors, particularly during periods of full financing by public healthcare systems. Our results reveal a correlation between the use of telerehabilitation services and the number of COVID-19 cases, suggesting that caregivers may prefer in-person services when epidemiological risk is low. This phenomenon may be attributed to various barriers and challenges hindering the widespread adoption of telerehabilitation, as identified by Rabanifar et al. (2022), including insufficient infrastructure, legal constraints, and lack of priority and determination. Additionally, clinical challenges, such as integrating TR technology into specific clinical environments, as highlighted by Schmeler et al. (2009), further complicate the provision of TR services.

However, telerehabilitation holds promise as a means to address regional disparities and enhance healthcare accessibility, aligning with the findings of Pruv Bettger et al. (2020) regarding telemedicine's potential to bridge healthcare gaps. Despite the challenges, telerehabilitation has demonstrated positive clinical outcomes comparable to conventional in-person approaches, as evidenced by studies conducted by Goyal and Kumar (2021) and Suso-Martí et al. (2021). Notably, research by Seron et al. (2021) suggests that telerehabilitation may offer comparable or even superior outcomes in various conditions, including osteoarthritis, low-back pain, hip and knee replacement, multiple sclerosis, and cardiac and pulmonary rehabilitation.

Furthermore, contrasting trends between telerehabilitation and teleconsultations in primary care were observed in the Polish healthcare context, as indicated by Furlepa et al. (2022). While teleconsultations surged in 2021 compared to 2020, our findings highlight the need for further investigation into the factors driving divergent trends between different healthcare sectors. Similarly, in the US, the COVID-19 pandemic led to an abrupt and subsequently sustained uptick in telemedicine utilization, with national telemedicine utilization peaking in April 2020 (Vogt et al., 2022). Overall, these insights contribute to a nuanced understanding of telerehabilitation's role in healthcare delivery, underscoring its potential to overcome barriers and enhance patient access to rehabilitation services.

## Conclusion

In conclusion, our study reveals a notable increase in the utilization of telehealth services across both rehabilitation and ambulatory care settings as COVID-19 cases surged. Share of the teleservices in the period October 2020-December 2022 was much higher in the ambulatory (average 7,8%) than the rehabilitation sector (average 0,16%). Although, the analysis showed a moderate relationship between COVID-19 incidence and telehealth utilization (Spearman's rho from 0.39 and 0.52). Our findings demonstrate no statistically significant difference in Spearman's rho values between ambulatory care and rehabilitation, indicating a similar strength of response to the pandemic waves. This suggests that both sectors reacted to the waves of COVID-19 cases, underscoring the importance of telehealth in maintaining healthcare accessibility during times of crisis.

## Declarations

Funding: This research received no specific grant from any funding agency in the public, commercial, or not-for-profit sectors.
